# An Original Ferroptosis-Related Gene Signature Effectively Predicts the Prognosis and Clinical Status for Colorectal Cancer Patients

**DOI:** 10.3389/fonc.2021.711776

**Published:** 2021-06-24

**Authors:** Yanfei Shao, Hongtao Jia, Ling Huang, Shuchun Li, Chenxing Wang, Batuer Aikemu, Guang Yang, Hiju Hong, Xiao Yang, Sen Zhang, Jing Sun, Minhua Zheng

**Affiliations:** ^1^ Department of General Surgery, Ruijin Hospital, Shanghai Jiao Tong University School of Medicine, Shanghai, China; ^2^ Shanghai Minimally Invasive Surgery Center, Ruijin Hospital, Shanghai Jiao Tong University School of Medicine, Shanghai, China; ^3^ Shanghai Institute of Digestive Surgery, Ruijin Hospital, Shanghai Jiao Tong University School of Medicine, Shanghai, China

**Keywords:** colorectal cancer, ferroptosis, gene signature, prognostic model, drug sensitivity

## Abstract

**Background:**

Colorectal cancer (CRC) is one of the most common malignant tumors in the world. Ferroptosis is a newly defined form of cell death, distinguished by different morphology, biochemistry, and genetics, and involved in CRC progression and treatment. This study aims to establish a predictive model to elucidate the relationship between ferroptosis and prognosis of CRC patients, to explore the potential value of ferroptosis in therapeutic options.

**Methods:**

The ferroptosis-related genes were obtained from the GeneCards and FerrDb websites. The limma R package was used to screen the differential ferroptosis-related genes (DEGs) in CRC from The Cancer Genome Atlas (TCGA) dataset. The least absolute shrinkage and selection operator (LASSO) and multivariate Cox regressions were to establish the 10-gene prognostic signature. The survival and receiver operating characteristic (ROC) curves were illustrated to evaluate the predictive effect of the signature. Besides, independent prognostic factors, downstream functional enrichment, drug sensitivity, somatic mutation status, and immune feature were analyzed. Moreover, all these conclusions were verified by using multiple datasets in International Cancer Genome Consortium (ICGC) and Gene Expression Omnibus (GEO).

**Results:**

Ten ferroptosis-related gene signature (TFAP2C, SLC39A8, NOS2, HAMP, GDF15, FDFT1, CDKN2A, ALOX12, AKR1C1, ATP6V1G2) was established to predict the prognosis of CRC patients by Lasso cox analysis, demonstrating a good performance on Receiver operating characteristic (ROC) and Kaplan–Meier (K–M) analyses. The CRC patients in the high- or low-risk group showed significantly different fractions of immune cells, such as macrophage cells and CD8+ T cells. Drug sensitivity and somatic mutation status like TP53 were also closely associated with the risk scores.

**Conclusions:**

In this study, we identified a novel ferroptosis-related 10-gene signature, which could effectively predict the prognosis and survival time of CRC patients, and provide meaningful clinical implications for targeted therapy or immunotherapy. Targeting ferroptosis is a good therapeutic option for CRC patients. Further studies are needed to reveal the underlying mechanisms of ferroptosis in CRC.

## Introduction

Colorectal cancer (CRC) is one of the most common malignant tumors in the world. According to the latest epidemiological analysis, there were more than 1,900,000 new cases of CRC in 2020 in the world, ranking third among men and second among women. There were more than 930,000 cases of CRC deaths, ranking third in terms of mortality, and the trend has been increasing year by year (1). The tumorigenesis and progression of CRC is a complex process involving multiple steps and genes. Studies have shown that colorectal cancer cells have special biological behaviors, showing strong proliferation ability, easy to relapse, and metastasis (2). Currently, comprehensive management for colorectal cancer, including surgical resection, neoadjuvant chemoradiotherapy, postoperative chemoradiotherapy, targeted therapy, and immunotherapy, has significantly improved the prognosis of CRC individuals (3). Nevertheless, the prognosis for individuals diagnosed with advanced CRC remains poor, especially for those with distant metastases (4, 5).

Meanwhile, the lack of reliable and accurate biomarkers remains a challenge for diagnosis, prognosis, and treatment options in CRC (6). At present, the clinical treatment and prognosis of CRC patients are based on the comprehensive evaluation of the TNM stage system (7). However, the tumor is in a complex status and even the same-stage individuals can show significant heterogeneity in prognosis and response to clinical treatment, such as right-hemi and left-hemi colon cancer patients (8). Therefore, it is crucial and meaningful to explore better methods and identify key molecular markers to accurately predict the prognosis and monitor the treatments of cancer individuals.

No matter in physiological or pathological conditions, cell death is an inevitable and complicated process. Unlike other types of regulated cell death (RCD), such as apoptosis, necrosis, and autophagy, ferroptosis is first reported in 2012 by Stockwell and is a newly defined form of RCD, characterized by iron overload, lipid reactive oxygen species (ROS) accumulation, and lipid peroxidation (9, 10). Cells undergoing ferroptosis demonstrate different morphological, biochemical, and genetic features, including ruptured cell membranes, reduced mitochondrial size, increased density of the mitochondrial membrane, and lack of chromatin condensation, etc (11). Ferroptosis is involved in various diseases, such as tissue ischemia, stroke, neurodegeneration, and tumor (12), which has attracted the attention of many scholars around the world in recent years. It is reported that ferroptosis is involved in several tumors: glioma (13), breast cancer (14, 15), lung cancer (15), hepatocellular carcinoma (16), diffuse large B-cell lymphoma (17), melanoma (18), and colorectal cancer (19). Many studies have shown that different types of cancer are vulnerable to ferroptosis. Moreover, therapy-resistant cancer cells and cancer cells with metastatic ability are sensitive to ferroptosis, indicating that targeting ferroptosis could be an alternative and promising approach to the current anticancer therapies (20). There are studies indicated ferroptosis-related genes were closely related to tumorigenesis, progression, treatment and prognosis of CRC, including p53, GPX4, SLC7A11, EGFR etc. (20–23). Recently, there are studies exploring the prognostic value of ferroptosis-related gene signature from public databases in several tumors, including uveal melanoma, lung cancer, hepatocellular carcinoma, pancreatic cancer, and glioma (15, 24–28). However, there are still few scientific and clinical studies on the correlation between CRC and ferroptosis, whether ferroptosis is correlated with CRC prognosis, and identification of the key ferroptosis-related genes in CRC progression remains largely unknown. Despite significant progress in CRC gene signatures, few have considered the use of ferroptosis-related gene characteristics to construct a prognostic signature in CRC.

In this study, we have screened the key genes related to ferroptosis in CRC from the public datasets and explore their underlying mechanisms in CRC. Then, by lasso cox analysis, we also established a prognostic multigene signature based on ferroptosis-related differentially expressed genes (DEGs). Interestingly, this signature was not only an independent clinical prognostic factor but could also accurately predict the clinical status of CRC patients, such as tumorigenesis and progression, drug resistance, somatic mutation, and immune function. Finally, this signature was also verified by multiple datasets in ICGC and GEO datasets, and the expression of which was checked in CRC tumor specimen.

## Materials and Methods

### Acquisition of Data

The gene expression and relevant clinical data were all obtained from the multiple public datasets. The Genotype-Tissue Expression (GTEx) database contains the transcriptional gene expression of 42 healthy human tissues. In this study, the RNA sequencing data of normal colon tissues were downloaded from this database. The Cancer Genome Atlas (TCGA) database covers almost all the genomic data and clinical data of cancer individuals. In this study, TCGA-COAD dataset was chosen as the training cohort because of its exhaustive information. Meanwhile, GTEx and TCGA database were all developed by the National Human Genome Research Institute in America and their technologies and platforms are same with each other. The International Cancer Genome Consortium (ICGC) database is similar to the TCGA database, except that the clinical data of some tumors are incomplete, such as CRC. In this study, the ICGC dataset was applied as the testing cohort. GEO database contains the gene expression and clinical data submitted by research institutions around the world. GSE39582 is a dataset with a large series of CRC individuals collected from French and the mRNA expression profiles were analyzed by Affymetrix U133plus2 chip. In similar, mRNA expression profiles were also analyzed by HG-U133Plus 2.0 expression arrays in GSE14333. CRC patients who were metastatic or recurrent and received modified FOLFOX6 or Cetuximab were respectively obtained in GSE19860 and GSE19862. The mRNA expression profiles of them were all analyzed by Human Genome Gene Chip arrays U133. GPL570 is the platform for GSE39582, GSE14333, GSE19860 and GSE19862. Besides, the mRNA expression profiles of 8 CRC individuals who had been tested for response to Cetuximab were analyzed by expression arrays in GSE56386. GPL13607 (Agilent-028004 SurePrint G3 Human GE 8x60K Microarray) is the platform for GSE56386. In this study, GSE14333 and GSE39582 were utilized as the testing cohorts and GSE56386, GSE19860 and GSE19862 were applied to test the drug resistance of CRC individuals. All the gene expression and corresponding clinical data of the GTEx, TCGA, and ICGC datasets were downloaded from the University of California Santa Cruz (UCSC, https://xenabrowser.net/datapages/). And the somatic mutation data of the TCGA and ICGC datasets were downloaded from their websites (https://portal.gdc.cancer.gov/, https://dcc.icgc.org/releases/current/Projects). Besides, all corresponding data of the GSE14333, GSE39582, GSE19860, GSE19862, and GSE56386 datasets were downloaded from the GEO website (https://www.ncbi.nlm.nih.gov/geo/). All datasets were independent of each other and performed the same preprocessing. Firstly, we combined the expression of all CRC ferroptosis-related gene with corresponding clinical data. Then we eliminated control samples in all datasets. Finally, we divided all colorectal cancer samples into 4 groups, TCGA training group (435 samples), ICGC testing group (414 samples), GSE14333 testing group (184 samples), and GSE39582 testing group (533 samples). And the GSE56386 (8 samples), GSE19860 (14 samples), and GSE19862 (29 samples) datasets were applied to analyze the drug-resistance in colorectal cancer. The baseline information of CRC individuals in the TCGA, ICGC, GSE14333, and GSE39582 datasets were presented in **Table 1**.

**Table 1:** The baseline characteristics of CRC patients in the multiple datasets.

Clinical Characteristics	TCGA cohort	GSE39582	ICGC cohort	GSE14333
	n=435	n=533	n=414	n=184
**Age (median, range)**	66.89 (31-90)	66.89 (22-97)	66.61 (31-90)	65.75 (26-92)
**Gender (%)**				
** Female**	204 (46.9%)	245 (46.0%)	198 (47.8%)	86 (46.7%)
** Male**	229 (52.6)	288 (54.0%)	216 (52.2%)	98 (53.3%)
** Unknown**	2 (0.5%)	0 (0%)	0 (0%)	0 (0%)
**M(%)**				
** M0**	321 (73.8%)	463 (86.9%)	NA	NA
** M1**	57 (13.1%)	50 (9.4%)	NA	NA
** Mx**	49 (11.3%)	1 (0.2%)	NA	NA
** Unknown**	8 (1.8%)	19 (3.5%)	NA	NA
**N(%)**				
** N0**	254 (58.4%)	291 (54.6%)	NA	NA
** N1**	99 (22.8%)	128 (24.0%)	NA	NA
** N2**	80 (18.3%)	86 (16.1%)	NA	NA
** N3**	0 (0%)	5 (0.9%)	NA	NA
** Unknown**	2 (0.5%)	23 (4.3%)	NA	NA
**Stage (%)**				
** Stage I**	73 (16.8%)	31 (5.8%)	NA	32 (17.4%)
** Stage II**	168 (38.6%)	257 (48.2%)	NA	78 (42.4%)
** Stage III**	124 (28.5%)	192 (36.0%)	NA	74 (40.2%)
** Stage IV**	57 (13.1%)	49 (9.2%)	NA	0 (0%)
** Unknown**	13 (3.0%)	4 (0.8%)	NA	0 (0%)
**Status**				
** Alive**	345 (79.3%)	370 (69.4%)	375 (90.6%)	150 (81.5%)
** Dead**	88 (20.2%)	163 (30.6%)	39 (9.4%)	34 (17.4%)
** Unknown**	2 (0.5%)	0 (0%)	0 (0%)	0 (0%)

NA, not available.

### Generation of Ferroptosis-Related Genes

A total of 245 ferroptosis-related genes in this article were obtained from the GeneCards (https://www.genecards.org/), FerrDb website (http://www.zhounan.org/ferrdb/), and the previously reported literature, searching by the keyword “ferroptosis”. FerrDb (29) is the first database that contains almost all reported ferroptosis regulators and markers in the world, releasing on December 31, 2019. GeneCards (30) is an integrative database from which comprehensive information about all predicted and annotated human genes can be obtained.

### Screening of DEGs

All colorectal cancer gene expression data from GTEx, TCGA, ICGC, and GEO datasets had been normalized by using the scale function in the dplyr R package. To exclude the influence of extreme values or outliers, we deleted genes without expression value. Finally, we extracted the gene expression data of normal colorectal samples (211 samples) and colorectal cancer samples (435 samples) from the GTEx and TCGA datasets. The batch effect was tested before further analysis by the sva R package. Then the expression of ferroptosis-related genes was extracted to facilitate subsequent difference analysis. The limma R package was used to identify the differentially expressed genes (DEGs) between the normal samples and the tumor samples, screening out the ferroptosis-related DEGs. To screen the possible function of ferroptosis-related DEGs in colorectal cancer, we used clusterProfiler R package to perform the Gene Ontology (GO) function and the Kyoto Encyclopedia of Genes and Genomes (KEGG) pathway enrichment analysis based on these DEGs. We also used the GSEA software (https://www.gsea-msigdb.org/gsea/login.jsp/) to analyze the significantly enriched pathways of these DEGs and visualize them by ggplot2 R package.

### Establishment of a Prognostic Ferroptosis-Related Gene Signature

In the training group, univariate cox analysis of OS (P < 0.05) was used to screen the survival-associated ferroptosis DEGs. Different from the normal Cox regression analyses, the least absolute shrinkage and selection operator (LASSO) Cox regression analysis is confirmed as a better method which can calculate the risk scores and reduce the risk of overfitting together (31–33). Thus, we used the LASSO Cox regression analysis to prevent the risk of overfitting and establish a scoring system for CRC individuals by the glmnet R package. Then, the regression coefficient of each gene and its corresponding mRNA expression to calculate the risk scores of the CRC individuals. The formula of risk score was “(regression coefficients × corresponding mRNA expression)”. Finally, the median score of the CRC individuals in the training group was used as a risk cut-off value to classify all CRC individuals into the high-risk or low-risk groups, including individuals in all testing groups (34, 35).

### Prediction Analysis

The survival curves between low-risk and high-risk groups in multiple datasets were tested by the Kaplan-Meier method. Besides, the time-dependent Receiver Operating Characteristic (ROC) curves and their areas under the ROC (AUC) curves were used to evaluate the predictive effect of the gene signature by survivalROC R package. Then, univariate Cox regression analysis was utilized to evaluate the predictive effect of different clinical characteristics between low-risk and high-risk groups in the TCGA datasets. Finally, principal component analysis (PCA) was applied to observe the clustering conditions of the prognostic signature, visualizing by the “scatterplot3d” R package. The survival curves (survival R package), the risk curves (pheatmap R package), the ferroptosis gene expression heatmaps (heatmap R package), and the ROC curve (survivalROC R package) of the training and testing groups were visualized by the related R package.

### Independent Prognostic Factors Analysis and Nomogram Prediction Model Construction

Univariate and multivariate prognostic analyses were performed to judge whether the risk score could be an independent prognostic factor in the TCGA training group and the GSE39582 testing group. Nomograms of the TCGA and GSE39582 group were established to predict the survival probability of CRC individuals in 1, 2, and 3 years, and the corresponding nomogram calibration curves were drawn based on the multivariate Cox regression analysis by the rms R package.

### Functional Analysis

Consistent with the method mentioned above, the limma R package was also used to screen the differentially expressed genes between the low-risk samples and the high-risk samples in the TCGA training group and the ICGC, GSE39582 testing groups. And the GO and KEGG analyses were performed by the clusterProfiler R package with the P < 0.05 and normalized enrichment score > 1. Testing the role of the prognostic signature in cetuximab, bevacizumab, and FOLFOX6 resistance by the GSE56386, GSE19860, and GSE19862 datasets. The maftools R package was applied to analyze and visualize the MAF files of somatic mutation data and calculate the tumor mutation burden(TMB) score of individuals in the TCGA and ICGC datasets.

### Immune Feature Analysis

TIMER is a comprehensive platform for analyzing the expression abundance of the six immune infiltration cells(dendritic cells, macrophages, neutrophils, CD8+ T cells, CD4+ T cells, and B cells) in malignant tumors. Based on the public resource, the associations between the signature and immune infiltrates were evaluated by Pearson correlation analysis and Student’s t-test. All the results were visualized by the ggplot2 R package. CIBERSORT is a novel deconvolution algorithm based on linear support vector regression. Considering the significant roles of the immune cells in the tumor microenvironment (TME), the scores of 22 immune cells in each tumor sample were calculated by CIBERSORT. All the results were shown as stacked graphs, heat maps, and box plots by ggplot2 R package. And the differential scores of these immune cells in TCGA, ICGC, and GSE39582 groups were tested by Wilcoxon rank-sum test.

### Expression Verification of the 10 Prognostic Ferroptosis-Related Genes in Datasets and CRC Specimen

We combined the gene expression data of control samples (17 samples) with those of CRC samples (533samples) in the GSE39582 group and performed the Wilcoxon rank-sum test to further compare the differential expression of the 10 prognostic ferroptosis-related genes between the normal and tumor colon tissues. Besides, a total of 75-paired normal/tumor CRC specimens were recruited from Ruijin Hospital (Shanghai, China) following the guidelines set by the Ethical Committee of Ruijin Hospital. The tumor and adjacent normal colon tissues were fixed by 10% formalin and embedded by paraffin. The optimum sections of tissue specimens were selected and deparaffinized and immunohistochemistry (IHC) was implemented as the following antibodies: HAMP (Abcam, ab30760), FDFT1 (Abcam, ab195046), GDF15 (Abcam, ab206414), TFAP2C (Abcam, ab218107).

### Statistical Analysis

The R software (version: 3.6.3) was utilized to conduct all the statistical analysis in this article. All P values of statistical data were based on two-sided statistical tests, and data with P < 0.05 was considered to be statistically significant.

## Results

### Identification and Functional Enrichment Analysis of Ferroptosis-Related DEGs

**Figure 1:**
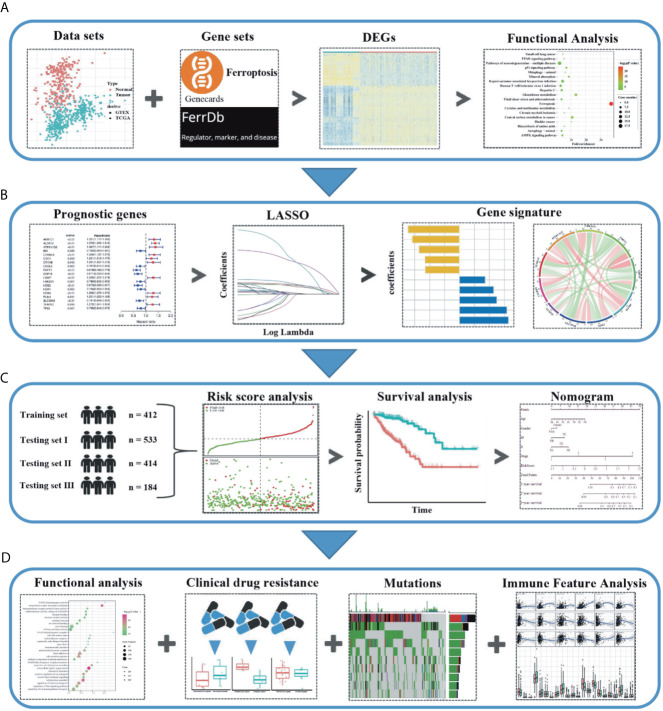
Schematic diagram of the study design. **(A)** The ferroptosis-related differentially expressed genes (DEGs) were identified between the tumor and normal colon tissues in the GTEx and TCGA datasets. **(B)** Based on these DEGs, the survival associated ferroptosis DEGs were identified in the TCGA dataset and the LASSO COX analysis was conducted to construct the ferroptosis-related gene signature. **(C)** Combined methods to evaluate and verify the predictive effect of the ferroptosis-related gene signature in the multiple datasets. **(D)** The relationship between the ferroptosis-related gene signature and the downstream functional enrichment, drug sensitivity, somatic mutation status, and immune feature in CRC patients.

**Figure 2:**
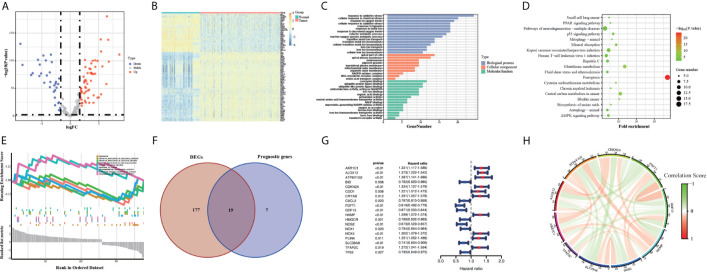
Volcano map **(A)** and Heatmap **(B)** of the ferroptosis DEGs in normal and tumor tissues from the GTEx and TCGA datasets. GO **(C)**, KEGG **(D)** and GSEA **(E)** analyses of the ferroptosis DEGs. Venn plot **(F)** to identify the DEGs that were correlated with OS. Forest plot **(G)** to show the results of the univariate cox regression analysis between DEGs expression and prognosis. The correlation network plot **(H)** of the 10 genes.

The flow chart of this study was developed in **Figure 1**. The batch inspection effect between the GTEx and TCGA datasets was perfect (**Figure S1A**) and an overwhelming majority of the ferroptosis-related genes (196/245, 80%) were differentially expressed between the 211 normal colorectal samples and 435 colorectal cancer samples by FDR < 0.01. The volcano plot displayed the difference in the expression of ferroptosis-related genes between the normal and tumor samples (**Figure 2A**) and the heatmap plot showed the differential expression of the top and low 30 ferroptosis-related genes based on the |log2FC| between the normal and tumor samples (**Figure 2B**). Then, 90 significant ferroptosis-related DEGs were further screened by FDR <0.01 and |log2FC| > 1 to perform the downstream functional enrichment analysis. Biological Process (BP), Molecular Function (MF), and Cell Component (CC) were all included in the GO function analysis and the results were shown in the bar plot (**Figure 2C**) with the P < 0.05 and normalized enrichment score > 1. The apical part of the cell was enriched for CC, response to oxidative stress for BP, coenzyme binding for MF. In addition, 19 KEGG pathways of these DEGs were enriched and shown in the bubble plot (**Figure 2D**). Besides Ferroptosis, the p53 signaling pathway, PPAR signaling pathway, and AMPK signaling pathway were also enriched, each of which was the classic pathway in CRC tumorigenesis. Finally, CELL_PROLIFERATION and PROTEIN_METABOLIC_PROCESS were enriched by the GSEA software and shown in the plot (**Figure 2E**).

### Construction of Ferroptosis-Related Prognostic Signature

First, we eliminated 23 samples without complete OS time information, which were necessary for the follow-up analysis in the TCGA training group. Then 26 survival-associated genes were screened from all the 245 ferroptosis-related genes by the univariate cox analysis of OS (P < 0.05) (**Figure S1B**). 19 of them were also ferroptosis-related DEGs, showing in the Venn plot (**Figure 2F**). According to the value of hazard ratio (HR), the genes (CDO1, CRYAB, PLIN4, NOX4, TFAP2C, HAMP, CDKN2A, ALOX12, AKR1C1, ATP6V1G2) were considered as the risk genes, while the genes (CXCL2, BID, HMGCR, TP53, NOX1, SLC39A8, NOS2, GDF15, FDFT1) as the protective genes (**Figure 2G**). To prevent the risk of over-fitting, the LASSO Cox regression analysis was utilized to construct a prognostic signature based on the expression of the 19 genes above. And a 10-gene signature (TFAP2C, SLC39A8, NOS2, HAMP, GDF15, FDFT1, CDKN2A, ALOX12, AKR1C1, ATP6V1G2) was filtered out by the minimum value of lambda (λ) (**Figures S1C, D**). The full names, functions, and coefficients of these genes were shown in **Table 2**. The correlation among these genes was shown by the circle plot (**Figure 2H**). Besides, the risk scores of the signature were used to predict prognosis in CRC individuals. By the formula mentioned in the above methods, the calculation of them were as follows: 0.1132 × expression of TFAP2C + (-0.0049) × expression of SLC39A8 + (-0.1244) × expression of NOS2 + 0.0361 × expression of HAMP + (-0.0913) × expression of GDF15 + (-0.1914) × expression of FDFT1 + 0.0684 × expression of CDKN2A + (0.2218) × expression of ALOX12 + 0.0845 × expression of AKR1C1 + (0.0923) × expression of ATP6V1G2.

**Table 2:** The information of the 10 genes in the multivariate Cox regression analysis.

Full Name	Function	Coefficient	HR	P value
Transcription Factor AP-2 Gamma	Sequence-specific DNA-binding protein that interacts with inducible viral and cellular enhancer elements to regulate transcription of selected genes	0.1132	1.272	0.019
Solute Carrier Family 39 Member 8	Electroneutral transporter of the plasma membrane mediating the cellular uptake of fe, zinc and manganese, three divalent metal cations important for development, tissue homeostasis or immunity	-0.0049	0.741	<0.01
Nitric Oxide Synthase 2	Produces nitric oxide (NO) which is a messenger molecule with diverse functions throughout the body	-0.1244	0.673	<0.01
Hepcidin Antimicrobial Peptide	Acts by promoting endocytosis and degradation of ferroportin, leading to the retention of iron in iron-exporting cells and decreased flow of iron into plasma	0.0361	1.299	<0.01
Growth Differentiation Factor 15	Regulates energy expenditure and body weight in response to metabolic and toxin-induced stresses	-0.0913	0.671	<0.01
Farnesyl-Diphosphate Farnesyltransferase 1	Catalyzes the condensation of 2 farnesyl pyrophosphate (FPP) moieties to form squalene and reduction with NADPH or NADH to form squalene	-0.1914	0.619	<0.01
Cyclin Dependent Kinase Inhibitor 2A	Capable of inducing cell cycle arrest in G1 and G2 phases, and Isoform smARF may be involved in regulation of autophagy and caspase-independent cell death	0.0684	1.334	<0.01
Arachidonate 12-Lipoxygenase	Plays a role in apoptotic process, promoting the survival of vascular smooth muscle cells for instance; May also play a role in the control of cell migration and proliferation	0.2218	1.373	<0.01
Aldo-Keto Reductase Family 1 Member C1	Converts progesterone to its inactive form, 20-alpha-dihydroxyprogesterone (20-alpha-OHP)	0.0845	1.331	<0.01
ATPase H+ Transporting V1 Subunit G2	Catalytic subunit of the peripheral V1 complex of vacuolar ATPase (V-ATPase); V-ATPase is responsible for acidifying a variety of intracellular compartments in eukaryotic cells	0.0923	1.387	<0.01

### Evaluation and Validation of Ferroptosis-Related Gene Signature

**Figure 3:**
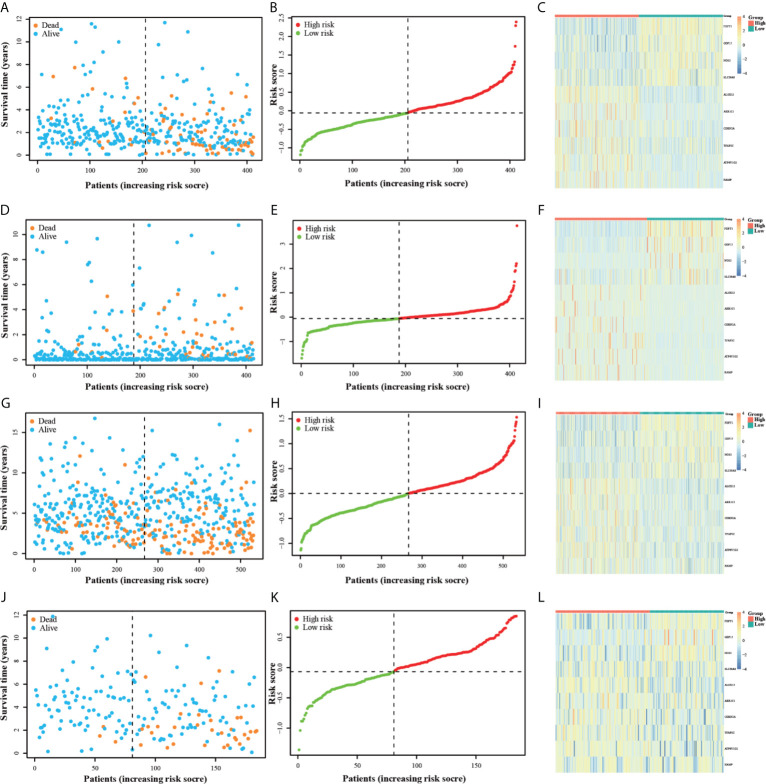
The OS status plots, OS and risk score plots and heatmaps of these 10 genes in the TCGA **(A–C)** ICGC **(D–F)** GSE14333 **(G–I)** and GSE39582 **(J–L)** datasets.

Individuals in all four datasets were classified into low-risk and high-risk groups based on the median value of risk scores in the TCGA training group. The scatterplots (**Figure 3A**) were drawn to show the distribution of risk scores and the correlation between risk scores and OS in the TCGA training group. The high-risk individuals had a higher probability to encounter death earlier than low-risk individuals from the **Figure 3B**. This result was also verified in the ICGC, GSE14333, and GSE39582 testing groups (**Figures 3D, E, G, H, J, K**). Heatmap (**Figure 3C**) was drawn to show the prognostic risk gene-expression profiles between the high-risk and low-risk TCGA training groups. Meanwhile, the same heatmaps (**Figures 3F, I, L**) were illustrated to display the prognostic risk gene-expression profiles of the three test groups. And there was no difference in the distribution of these prognostic risk gene-expression profiles between the training and testing groups.

**Figure 4:**
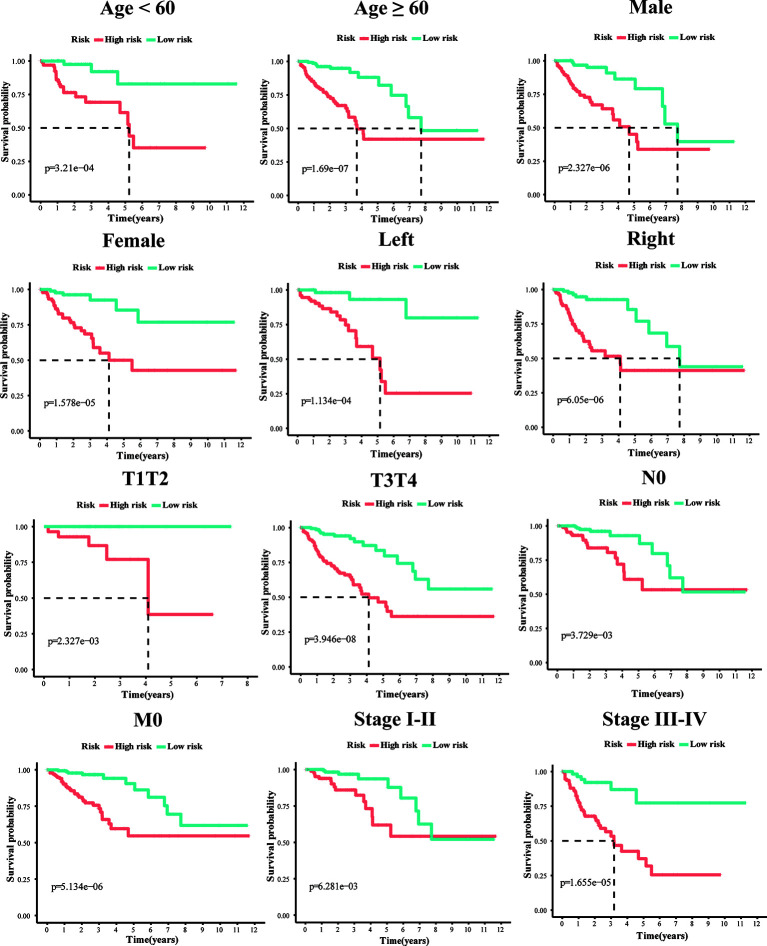
Kaplan–Meier survival plots of the 10 gene signature in TCGA dataset with different clinical characteristics (Age, Gender, T1+T2, T3+T4, N0, M0, stage I+II, stage III+IV, Left-hemi CRC and Right-hemi CRC).

To explore whether the 10-gene signature could be widely used to determine the survival conditions in different clinical characteristics, the stratification analysis was used in the age (≤60 and >60), gender (male and female), location (left-hemi and right-hemi), M stage, N stage, and TNM stage. The results indicated that individuals in the low-risk group had significantly better OS than individuals in the high-risk group for each subgroup (P < 0.01, **Figure 4**).

**Figure 5:**
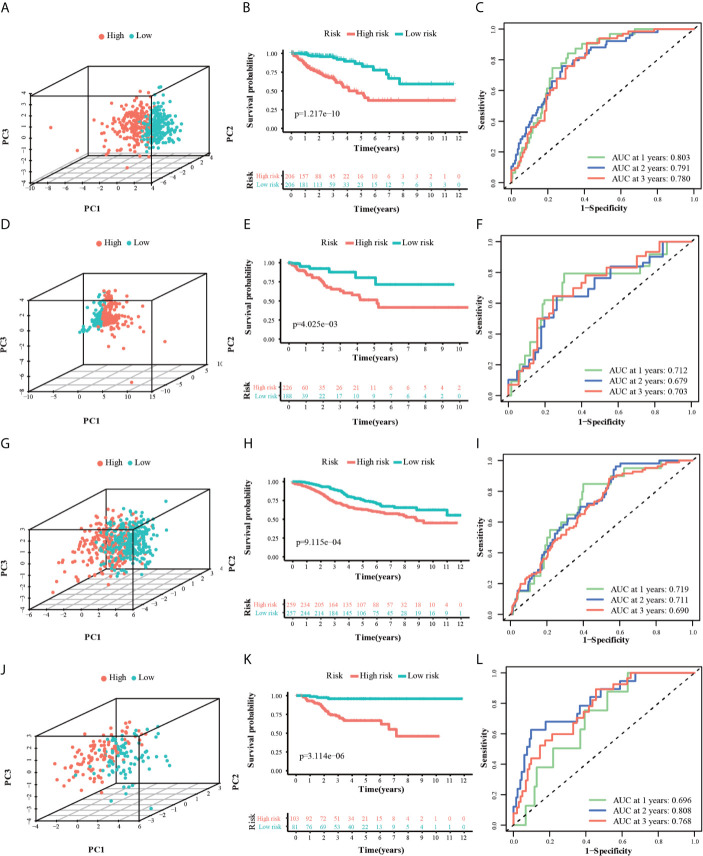
3dPCA plots of the TCGA **(A)**, ICGC **(D)**, GSE14333 **(G)** and GSE39582 **(J)** datasets. Kaplan-Meier curves for the OS of patients in the high-risk group and low-risk group in the TCGA **(B)**, ICGC **(E)**, GSE14333 **(H)** and GSE39582 **(K)** datasets. AUC of time-dependent ROC curves for the risk score in the TCGA **(C)**, ICGC **(F)**, GSE14333 **(I)** and GSE39582 **(L)** datasets.

PCA analysis revealed that the individuals in different risk levels could be distributed into two sections (**Figures 5A, D, G, J**). The survival curves (**Figure 5B**) indicated a higher probability of survival in the low-risk TCGA training group (P < 0.0001). The ROC curves (**Figure 5C**) evaluated the predictive effects of the risk score for OS, and the area under the curve (AUC) of the 10-gene signature reached 0.803 (1 year), 0.791 (2 years), and 0.780 (3 years), illustrating a strong separation capability. This result was verified in the other three test groups and the details were as follows. In the ICGC, GSE14333, and GSE39582 testing groups, high-risk individuals were also more likely to have a reduced OS time compared with low-risk individuals (**Figures 5E, H, K**, P < 0.001). Besides, their respective AUC all reached around 0.70 and the minimum AUC among them could reach 0.678 (**Figures 5F, I, L**). Furthermore, we divided the GSE14333 testing group, which consisted of pTNM II–III colorectal cancer individuals, into the left-hemi colon cancer and right-hemi colon cancer groups. It was noteworthy that the 10-gene signature also performed well both in the left-hemi colon cancer (**Figures S2A–E**, P < 0.0001, maximum AUC = 0.835) and right-hemi colon cancer groups (**Figures S2F–L**, P = 0.042, maximum AUC = 0.643). These results above indicated that the 10-gene signature could provide an effective prognostic value in CRC individuals.

### Independent Prognostic Factors Analysis and Nomogram Prediction Model Construction

**Figure 6:**
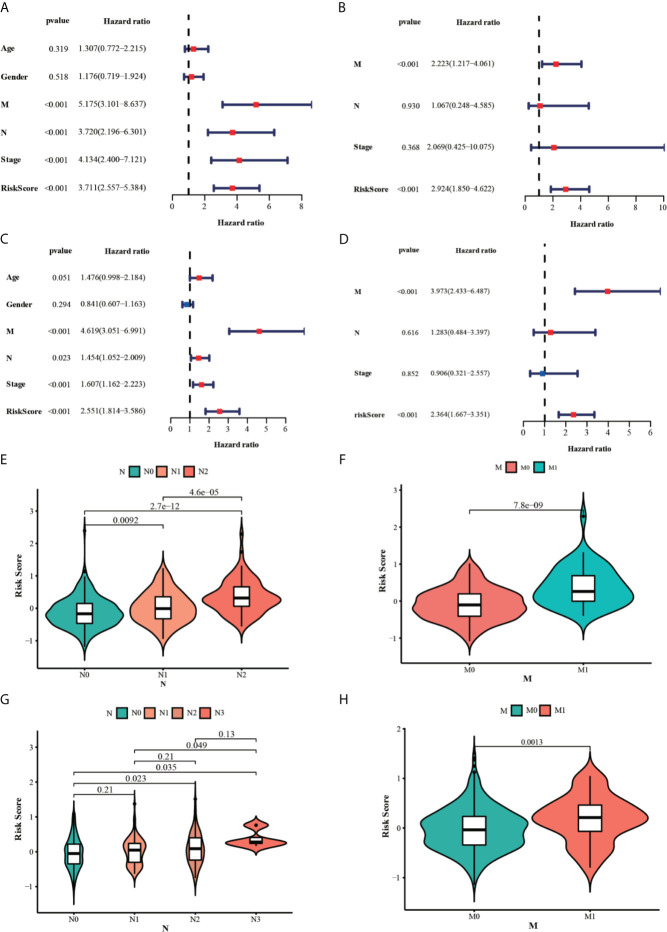
Results of the univariate and multivariate Cox regression analyses regarding OS in the TCGA **(A, B)** and the GSE39582 **(C, D)** datasets. The violin plots for the relationships between the risk scores and the clinical indicators (M and N stage) in the TCGA **(E, F)** and the GSE39582 **(G, H)** datasets.

**Figure 7:**
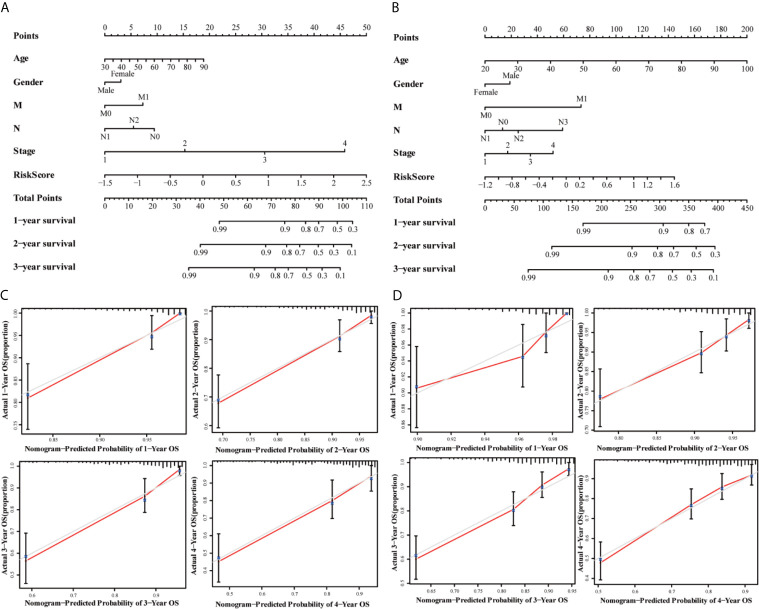
Nomograms for predicting 3-year survival in the TCGA **(A)** and GSE39582 **(B)** datasets. Calibration curves for the nomogram predicting 1-4 years survival in the TCGA **(C)** and GSE39582 **(D)** datasets.

The univariate and multivariate cox regression analyses were performed to evaluate the prognostic value of the risk score. The univariate Cox regression analysis indicated that the risk score (P < 0.001, HR = 3.711, 95% CI = 2.557–5.384) and related clinical parameters, including M stage (P < 0.001, HR = 5.175, 95%CI = 3.101–8.637), N stage (P < 0.001, HR = 3.720, 95%CI = 2.196–6.301), and TNM stage (P < 0.001, HR = 4.134, 95%CI = 2.400–7.121), were significantly associated with OS in the TCGA training group (**Figure 6A**). Multivariate Cox regression analysis revealed that the risk score (P < 0.001, HR = 3.711, 95% CI = 2.557–5.384) and M stage (P < 0.001, HR = 3.639, 95%CI = 2.510–5.277) were the independent prognostic factors of the OS (**Figure 6B**). Besides, the risk score was also correlated with clinical parameters in the GSE39582 testing group. Similar to the results obtained from the TCGA training group, the risk score (P < 0.001, HR = 2.314, 95% CI = 1.603–3.340) in the GSE39582 testing group were also proven to be the independent prognostic factors of the OS (**Figures 6C, D**). In addition, CRC individuals at the M1 stage in TCGA training and GSE39582 testing groups all had higher risk scores than those at the M0 stage (P < 0.01, **Figures 6F, H**). The same results were confirmed in the N stage (P < 0.05, **Figures 6E, G**). The clinical characteristics of Age, Gender, M, N, Stage, and Risk Score were together used to construct the nomogram prediction models in TCGA and GSE39582 groups (**Figures 7A, B**) to predict the survival probability of CRC individuals at 1, 2, and 3 years. The calibration curves were shown to indicate the great prediction in the actual observations in 1-4 years (**Figures 7C, D**).

### Functional Enrichment Analysis of Ferroptosis-Related Gene Signature

**Figure 8:**
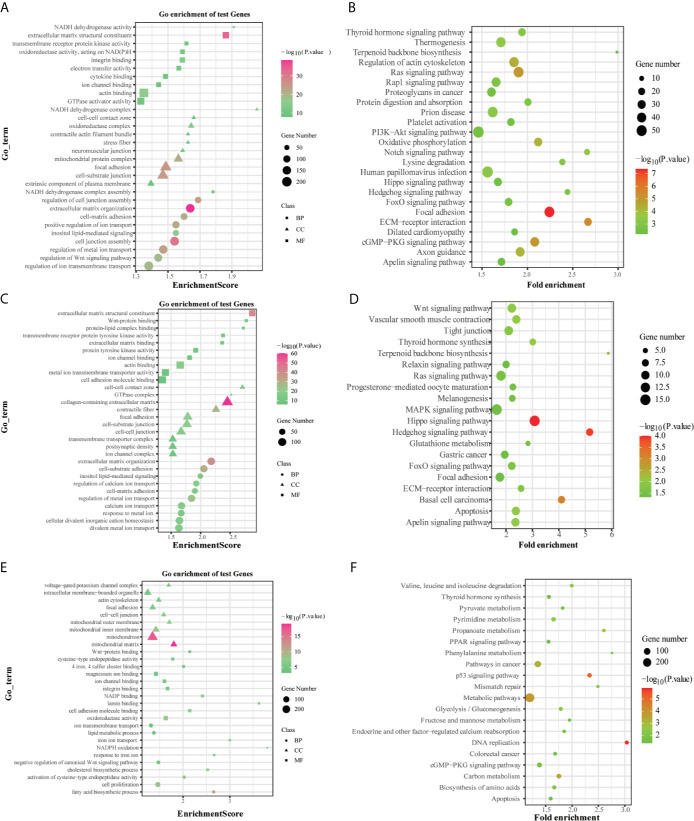
GO and KEGG analyses of the DEGs between the high-risk and low-risk groups in the TCGA **(A, B)**, ICGC **(C, D)** and GSE39582 **(E, F)** datasets.

The GO enrichment and KEGG pathway analysis performed by the DEGs between the high-risk and low-risk groups were utilized to explore the biological functions and pathways that were related to the risk score. The GO enrichment analysis results showed that DEGs were enriched in several iron-related molecular functions, such as ion channel activity and ion gated channel activity in the training and testing groups (P. adjust < 0.05, **Figures 8A, C, E**). Interestingly, the DEGs from the training and testing groups were all enriched in several tumor metastasis-related biological processes, such as cell junction, cell adhesion, and cell-cell junction (P. adjust < 0.05). Meanwhile, 7 metastasis-related pathways were enriched in the TCGA training group based on KEGG pathway analysis, including Regulation of actin cytoskeleton, PI3K-Akt signaling pathway, Notch signaling pathway, FoxO signaling pathway, Focal adhesion, ECM-receptor interaction, cGMP-PKG signaling pathway (P. adjust < 0.05). And the FoxO signaling pathway, Focal adhesion, ECM-receptor interaction pathway, and cGMP-PKG signaling pathway were also enriched in the testing groups (P. adjust < 0.05, **Figures 8B, D, F**). To summarize, these results mentioned above suggested that the risk score of the 10-gene signature was closely related to tumorigenesis and metastasis of CRC.

### The Relationship Between Ferroptosis-Related Gene Signature and Clinical Drug Resistance

**Figure 9:**
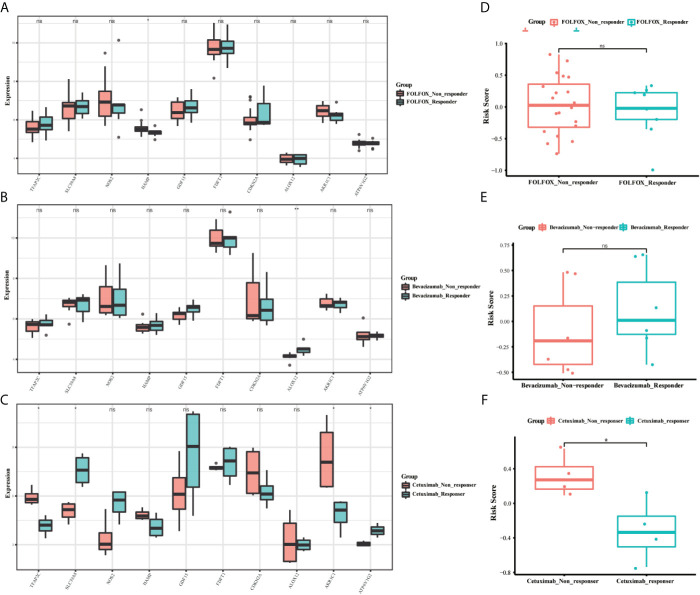
The boxplots of the different expression levels of the 10 ferroptosis-related genes between the FOLFOX **(A)**, Bevacizumab **(B)**, and Cetuximab **(C)** responders and non-responders in the GSE19860, GSE19862 and GSE56386 datasets. The boxplots for the relationships between the risk scores and the drug resistance in the GSE19860 **(D)**, GSE19862 **(E)** and GSE56386 **(F)** datasets. *p < 0.05, **p < 0.01. ns, no significance.

To identify whether the 10-gene signature was related to CRC resistance, the gene expression data of cetuximab-resistance, bevacizumab-resistance, and FOLFOX6-resistance were obtained from the GSE56386, GSE19860, and GSE19862 datasets. The box plots showed the expression distribution of the 10 genes in the three drug-resistance datasets (**Figures 9A–C**). There was a significant difference in the expression distribution of TFAP2C, SLC39A8, and AKR1C1 genes between the cetuximab responders and non-responders in colorectal cancer (P < 0.05). Furthermore, the risk scores of these individuals were calculated by the same formula to predict drug sensitivity. As expected, the CRC patients who did not respond to cetuximab had higher risk scores (P < 0.05) (**Figures 9D–F**). Therefore, we believed that the 10-gene signature could predict drug resistance in CRC patients.

### Gene Mutation in the Ferroptosis-Related Gene Signature

**Figure 10:**
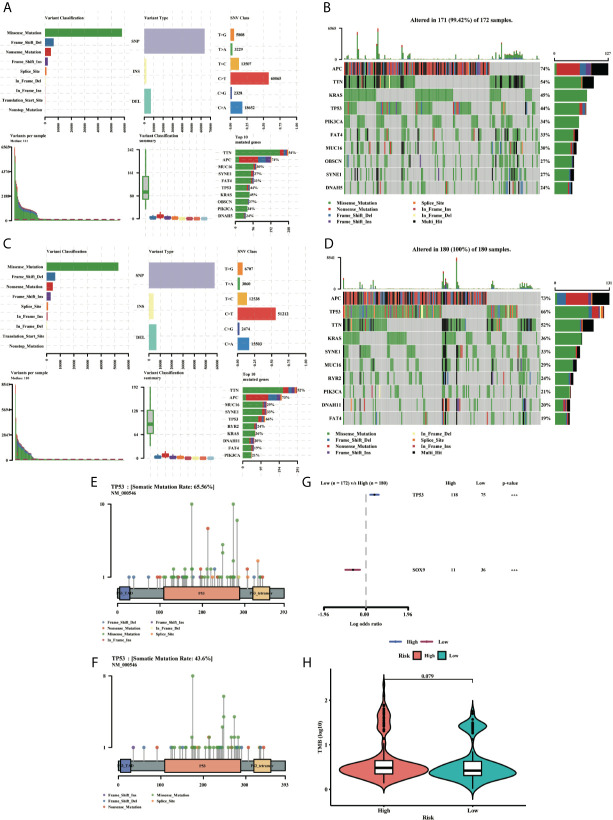
MAF-summary plots and oncoplots of the somatic mutation between the high-risk **(A, B)** and low-risk **(C, D)** groups in the TCGA dataset. Lollipop charts of the mutated TP53 gene in the high-risk **(E)** and low-risk **(F)** groups. Forest plot **(G)** of the differentially somatic mutation and the violin plot **(H)** for the TMB scores between the high-risk and low-risk groups.

To clarify whether there was a difference in gene mutation between high-risk and low-risk groups, we downloaded and analyzed simple nucleotide variation data from TCGA. The summaries of the gene mutation information were shown in the bar plot (**Figures 10A–D**). APC (73%), TP53 (66%), TTN (52%), KRAS (36%), and SYNE1 (33%) were the top five genes with the highest mutation frequencies in the high-risk group, and APC (74%), TTN (54%), KRAS (45%), TP53 (44%) and PIK3CA (34%) in the low-risk group, while TP53 was relatively high mutated and PIK3CA was low mutated in the high-risk group. The different mutation distributions of TP53 between the high-risk and low-risk groups were shown in the lollipop plot (**Figures 10E, F**). The same results were verified in the ICGC testing group (**Figures S3A–D**). Then, TP53 was also highly mutated in the high-risk group as compared to the low-risk group (**Figure 10G**). Besides, the Tumor Mutation Burden (TMB) was checked in both groups, indicating the TMB was not a significant variate, but with an increased tendency in the high-risk group in TCGA (**Figure 10H**), while the TMB level was higher with a significant difference in high-risk group in ICGC database (**Figure S3E**).

### Immune Feature Analysis of Ferroptosis-Related Gene Signature

**Figure 11:**
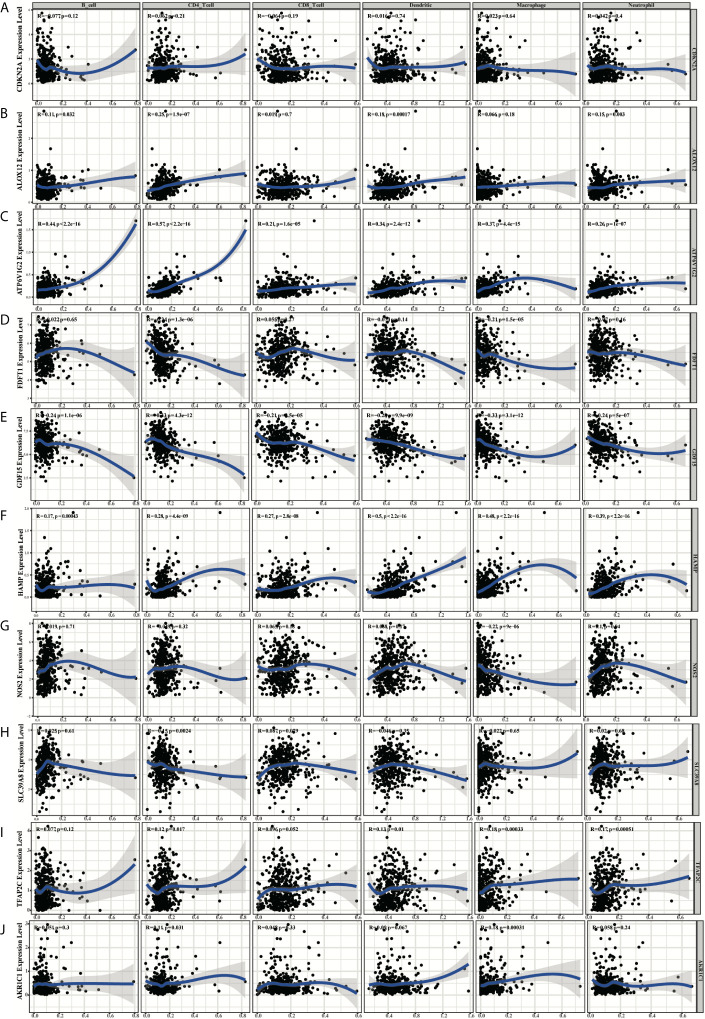
The diagrams of the correlation analysis between CDKN2A **(A)**, ALOX12 **(B)**, ATP6V1G2 **(C)**, FDFT1 **(D)**, GDF15 **(E)**, HAMP **(F)**, NOS2 **(G)**, SLC39A8 **(H)**, TFAP2C **(I)**, AKR1C1 **(J)** and the immune infiltration level in the TCGA dataset.

**Figure 12:**
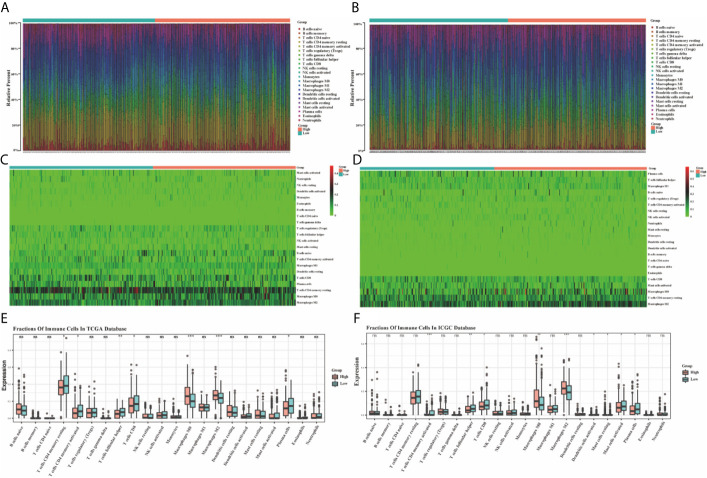
The bar graphs for the distribution of 22 tumor-infiltrating immune cells between the high-risk and low-risk groups in the TCGA **(A)** and ICGC **(B)** datasets. The heatmaps for the correlation between risk groups and 22 immune cells in the TCGA **(C)** and ICGC **(D)** datasets. The boxplots for the comparison of the 22 immune cells between the high-risk and low-risk groups in the TCGA **(E)** and ICGC **(F)** datasets. *p < 0.05, **p < 0.01, ***p < 0.001. ns, no significance.

Based on the TIMER, the relationships between the 10 prognostic ferroptosis-related genes and six immune infiltration cells were evaluated and the results indicated that most of these 10 genes were associated with these immune infiltration cells, especially TFAP2C, HAMP, GDF15, FDFT1, and ATP6V1G2 genes (**Figure 11**). Besides, the CIBERSORT method was also applied to estimate the different scores of 22 immune cells between high-risk and low-risk groups in the training and testing groups. The differences scores of 22 immune infiltration cells were shown in stacked graphs (**Figures 12A, B**). The different expressions of immune cells between the high-risk and low-risk were shown in the heat maps (**Figures 12C, D**). Finally, the results also indicated that macrophages M0 and macrophages M2 were down-regulated in the low-risk groups, while T cells CD4 memory activated, T cells follicular helper, T cells CD8, and Plasma cells were significantly up-regulated (p < 0.05, **Figures 12E, F**).

### Validation of the Expression Levels of the 10 Ferroptosis-Related Genes

**Figure 13:**
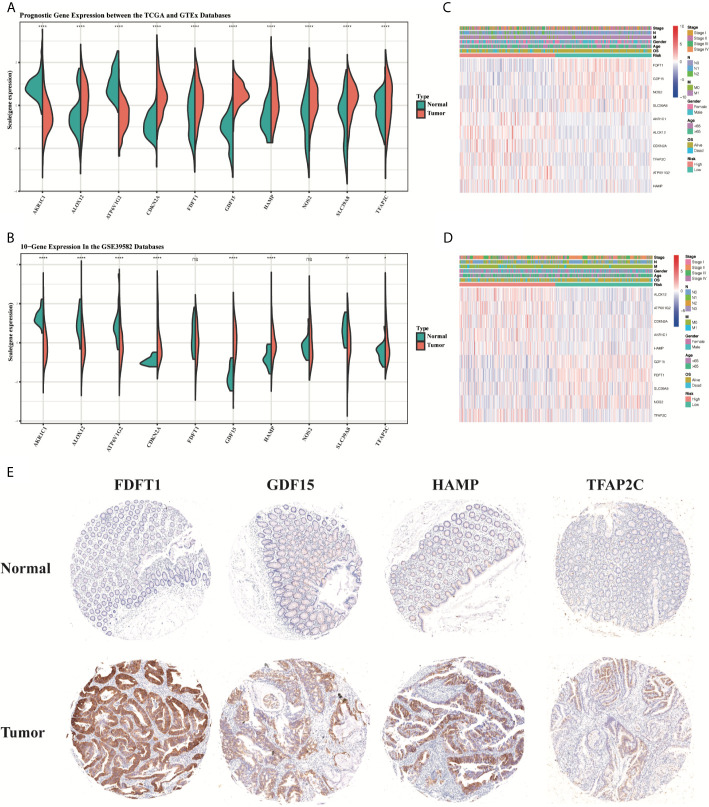
The boxplots of the different expression levels of the 10 ferroptosis-related genes between the normal and tumor colon tissues in the training **(A)** and testing **(B)** datasets. The heatmaps for the different expression levels of these 10 genes and the clinical characteristics between the high-risk and low-risk groups in the TCGA **(C)** and GSE39582 **(D)** datasets. Representative immunohistochemistry images **(E)** of FDFT1, GDF15, HAMP, and TFAP2C in CRC tissues and corresponding normal tissues. *p < 0.05, **p < 0.01, ****p < 0.0001. ns, no significance.

Compared to normal tissues, the expression of TFAP2C, SLC39A8, NOS2, HAMP, GDF15, FDFT1, CDKN2A, and ALOX12 was significantly upregulated, while the expression of AKR1C1 and ATP6V1G2 was downregulated in CRC tumor tissues in the TCGA training group (P < 0.05, **Figure 13A**). Most results obtained in GSE39582 testing group were similar to that in the training group, except the gene expression of ALOX12, FDFT1 and NOS2 (P < 0.05, **Figure 13B**). Then, the heatmaps for the different expression levels of these 10 genes and the clinical characteristics between the high-risk and low-risk groups were also drawn to describe their relationships (**Figures 13C, D**). Besides, the expression of HAMP, FDFT1, GDF15, TFAP2C was tested in our CRC tissue array, containing 75-paired CRC tissues. The results showed that the expression of HAMP, FDFT1, GDF15, TFAP2C all increased in tumor tissues, indicating their meaningful regulatory roles in CRC (**Figure 13E**).

## Discussion

The CRC patients, especially the ones at the advanced stage, have benefited a lot from the chemotherapy, targeted therapy, and immunotherapy, all of which were clinically adopted based on individual genomic or molecular signatures and specific tumor location (36, 37). Cetuximab, targeting epidermal growth factor receptor (EGFR), is widely accepted for metastatic CRC patients. However, only left-hemi CRC patients with KRAS/BRAF/PIK3CA wild-type statue could benefit from this targeted therapy (38, 39). Meanwhile, the inhibitors targeting the BRAF V600E mutations could improve the prognosis of CRC patients with this mutation (40). The latest KEYNOTE-177 study in 2021 demonstrated that Pembrolizumab (PD-1 inhibitor) could significantly improve the progression-free survival (PFS) of mCRC patients with MSI-H/dMMR for the first-line treatment (41). These results suggested CRC might have more complex mechanisms, further studies and new research fields are needed to fully understand underlying features of CRC.

Due to higher demand for cell proliferation and progression, cancer cells bear more active iron metabolism and higher ROS level than normal cells, especially for cells under EMT process with more invasive abilities (10, 42). Indeed, these cancer cells don’t undergo ferroptosis or uncontrolled oxidative stress because they can adjust several defense mechanisms, mainly including an increase in antioxidant mechanisms to remove excessive ROS and ensure the proliferation and progression of cancer cells. Just because of this vital reliance, cancer cells become vulnerable to ferroptosis, a new form of cell death characterized by an iron-dependent accumulation of lipid peroxidation, induced by antioxidant turbulence (10). However, there is rare study concerned with the prognostic value of ferroptosis in CRC. Therefore, it’s necessary to identify the key ferroptosis-related genes in CRC, clarify the relationship between ferroptosis and CRC. In this study, we have collected 260 ferroptosis-related genes reported in public, systematically analyze their expression in normal and tumor tissues of colorectal cancer. As expected, 196 (80%) ferroptosis-related DEGs were identified after screening conditions were adjusted to FDR <0.01, suggesting ferroptosis plays a significant role in CRC. And 19 ferroptosis-related DEGs were further confirmed to be significantly correlated with the CRC individuals’ OS. Then using the lasso cox analysis, a novel prognostic 10-gene signature was constructed and externally validated in several external datasets, including TFAP2C, SLC39A8, NOS2, HAMP, GDF15, FDFT1, CDKN2A, ALOX12, AKR1C1, ATP6V1G2.

To further elucidate the role of these ten genes in CRC, we analyzed their mRNA expression levels and summarized their main molecular functions. According to the value of hazard ratio (HR), TFAP2C, HAMP, CDKN2A, ALOX12, AKR1C1, and ATP6V1G2 were considered as the risk genes, while SLC39A8, NOS2, GDF15, FDFT1 as the protective genes. TFAP2C, a transcription factor in ferroptosis, plays an important role in regulating ferroptosis sensitivity through transcription mechanisms and modulates ferroptosis in gallbladder cancer through the Nrf2 signaling pathway (43). SLC39A8 has been reported as an important membrane transporter in iron metabolism, differentially expressed in colons, and was associated in CRC tumor aggressiveness (44, 45). NOS2 is a key gene in oxidative metabolism, and consistent with our results, Miteva LD’s team also found that the expression of NOS2 was significantly downregulated in advanced CRC individuals, especially in metastasis individuals (46). HAMP is a negative regulator of ferroptosis which can export iron (47). The gene was widely expressed in colorectal cancer tissue and correlated with ferroportin repression (48). GDF15 has been reported to be a pivotal regulator of tumor cell invasion and metastasis and could promote ferroptosis in tumor cells (49). FDFT1 plays an important role in cholesterol biosynthesis and ferroptosis (50). FDFT1 knockdown is correlated with malignant progression and poor prognosis in CRC and inhibits tumorigenesis by negatively regulating AKT/mTOR/HIF1α signaling pathway (51). CDKN2A can induce cell cycle arrest in G1/G2 phase and play a key role in tumorigenesis by enhancing p53-dependent transactivation and ferroptosis (52). ALOX12 has been reported to be the core enzyme in mediating lipid peroxidation and has significant correlation with glutathione peroxidase 4 (GPX4) (53). AKR1C1 protects cancer cells from ferroptosis and is considered as the ferroptosis-protective gene (54). In addition, AKR1C1 has been confirmed high basal expression levels in CRC and promotes tumor proliferation, metastasis, and drug resistance (55). ATP6V1G2, involved in autophagy, apoptosis and necrosis, plays a significant role in human energy metabolism and induces oxidative stress, which is an important intermediate process of ferroptosis (49). In general, based on their molecular functions, these ten genes could be divided into four categories, including transcription factors (TFAP2C), energy metabolism (GDF15, FDFT1, AKR1C1, ATP6V1G2), iron metabolism (SLC39A8, HAMP, CDKN2A), and oxidative metabolism (NOS2, ALOX12).

Based on this 10-gene signature, CRC patients in the high-risk group were found to have a significantly shorter OS in the training and other three testing groups. Furthermore, this signature was also tested in right-hemi and left-hemi colon cancer in GSE14333 due to their specific molecular features, demonstrating similar and effective prognostic value. The multivariate cox regression analyses also demonstrated that the signature-based risk score was an independent predictor of OS. Besides, we constructed a nomogram by integrating the age, gender, stage, N stage, M stage, and risk scores of this signature, offering a reliable and easy approach for clinicians to predict the survival of CRC patients. Recently, Ubellacker et al. (56) reported that tumor cells in lymph nodes experienced less oxidative stress and showed resistance to ferroptosis, resulting in an increased probability of lymph node seeding and the ability to colonize distant organs. In other words, lymph nodes may protect tumor cells from ferroptosis. Their study, providing new insights into tumor metastasis, has been widely accepted by other teams (57). In our study, we got similar results: there was a significant difference between the patients with lymph node metastasis (N1+N2) and patients without lymph node metastasis (N0) based on this 10-gene signature, and N2 patients had higher risk scores than N1 and N0 patients. The risk scores were also significantly higher in patients with metastasis (M1) than in non-metastasis (M0). Meanwhile, our functional analyses based on the DEGs in the high-risk and low-risk patients, showed that PI3K-Akt signaling pathway, Focal adhesion, ECM-receptor interaction, Notch signaling pathway and Cell matrix adhesion, etc. were enriched, all of which were closely involved in the metastasis process. Meng Wang et al. also demonstrated Gambogenic acid-induced-ferroptosis could inhibit the EMT process in melanoma cells (58). Neratinib, a pan-tyrosine kinase inhibitor, was reported to potently inhibited tumor growth and metastasis in breast cancer. All of these studies indicated ferroptosis is closely related to tumor metastasis (59).

As mentioned above, targeted therapy and chemotherapy have improved the survival of CRC patients. We checked the potential value of ferroptosis-related signatures in the response to common drugs in different databases. Surprisingly, the patients in the high-risk group showed resistance to cetuximab, bevacizumab, and FOLFOX6, and this trend was significant in the cetuximab group. Peng Chen et al. reported that combinative treatment of cetuximab and β-elemene, a natural product, is sensitive to KRAS mutant colorectal cancer cells by inducing ferroptosis (20). The study from Annalisa Lorenzato’s team demonstrated that Vitamin C limits the acquired resistance to cetuximab through disrupting iron homeostasis and inducing ferroptosis (19). These results suggested ferroptosis-related signature could be a potential indicator for targeted therapy.

The tumor suppressor protein p53 (TP53), recognized as “the guardian of the genome”, plays a critical role in the normal and pathological process (60). Besides apoptosis, cell cycle, and autophagy, ferroptosis is also tightly regulated by TP53 (53, 61). However, TP53 demonstrated a bidirectional effect on ferroptosis depending on the specific microenvironment (60). TP53 could induce ferroptosis through inhibiting SLC7A11 (61), enhancing SAT1 (62) and GLS2 (63). On the contrary, TP53 inhibited ferroptosis by suppressing DPP4 activity (23) and increasing p21 expression (64). In our study, TP53 was identified among the 19-prognostic DEGs in CRC, showing a significant role during ferroptosis. More importantly, there was a higher frequency of TP53 mutation in the high-risk group. It was reported that TP53^3KR^ mutant, not p53^4KR98^ and other N-terminal mutants, could induce ferroptosis, but unable to affect the cell cycle and apoptosis (60, 61). It’s possible that ferroptosis inhibition in pretumor cells by TP53 mutation induces tumorigenesis (26). Further studies are needed to reveal the specific mechanisms of TP53 in the ferroptosis of CRC.

Cell death and immunity are recognized as the two evolutionary conserved processes to keep the homeostasis of the body. The roles of ferroptosis in the tumor immune microenvironment are still not clear. Our study demonstrated CRC patients with high-risk scores or in the high-risk group harboring obvious immune-suppressive features: there was a higher infiltration of M0 Macrophages, and M2 Macrophages and lower infiltration of CD8+ T cells and CD4+ memory T cells in the high-risk group, indicating ferroptosis was involved in tumor immune microenvironment and a stronger immunosuppressive effect contributed to poor survival of CRC patients in the high-risk group. Immune checkpoint blockade therapy is one of the most successful and important progress for anti-cancer immunotherapies through activating the natural tumor-selective killing activity of T cells. Wang et al. demonstrated that ferroptosis could mediate the anti-tumor activity of CD8+ T cells in response to PD-L1 blockade, further study showed that interferon-gamma secreted by CD8+ T cells could inhibit the expression of system xc-, a key regulator of ferroptosis, to induce ferroptosis of tumor cells (65). Colorectal cancer (CRC) infiltration by cells expressing CD8 positive T lymphocytes has been shown to be independently associated with favorable prognosis (66, 67). TMB is defined as the total number of somatic coding mutations, high risk scores were positively correlated with TMB, which predicting good outcomes of immune checkpoint inhibitor treatments (68). Besides, PD1 expression was also different between high and low-risk groups (**Figure S4**). These results indicated that patients in the high-risk group could be sensitive to immunotherapy, or checkpoint inhibitor-based immunotherapy. Immunotherapy combined with ferroptosis-targeting therapy might be a feasible therapeutic approach. Almost at the same time with our work, there were some studies reported the prognostic signature of ferroptosis-related genes for colon cancer patients (69, 70). Compared to their works, this signature in our study demonstrated better predictive ability. Besides, we further addressed the possible mechanisms and potential value of this signature in clinical treatments.

Our study had the following strengths. First, we systemically analyzed the expression ferroptosis-related gene in CRC, constructed and validated a reliable ferroptosis-related gene signature to predict the prognosis of CRC patients by various datasets. Second, we demonstrated the potential clinical value of this signature in drug-resistance, immunotherapy, and predicting patient survival time. Third, this signature may have a good predictive value in other cancers, further validation will be needed. Besides, this study has several limitations. First, the retrospective data from several databases was adopted in this signature, and further prospective studies are needed to testify its clinical value. Second, we only analyzed four genes expression in CRC samples of our center, more gene expression and their relationship with clinical features of CRC patients should be studied. Third, underlying mechanisms of ferroptosis should be revealed in the future.

## Conclusion

Collectively, our study provided a good prognostic risk model for CRC patients. The reliability and effectiveness of this signature were validated in several datasets and clinical specimens. Importantly, this signature was also involved in the clinical treatments, including target therapy or immunotherapy, bringing us new insights into the underlying molecular mechanisms and the discovery of new treatments for CRC.

## Data Availability Statement

The original contributions presented in the study are included in the article/**Supplementary Material**. Further inquiries can be directed to the corresponding authors.

## Ethics Statement

The studies involving human participants were reviewed and approved by Ethical Committee of Ruijin Hospital. The patients/participants provided their written informed consent to participate in this study.

## Author Contributions

Conceptualization, SZ and JS. Methodology, YS and SZ. Software, YS. Validation, HJ, XY, and LH. Formal analysis, XY. Investigation, CW. Resources, BA. Data curation, GY and SL. Writing—original draft preparation, YS and SZ. Writing—review and editing, JS and MZ. Visualization, HH. Supervision, JS. Project administration, XY****. Funding acquisition, SZ and JS. All authors have read and agreed to the published version of the manuscript. All authors contributed to manuscript revision, read, and approved the submitted version.

## Funding

This research was funded by Youth Cultivation Project of Ruijin Hospital (Grant No. KY2021611) and the National Nature Science Foundation of China (NSFC) (Grant No. 81871984).

## Conflict of Interest

The authors declare that the research was conducted in the absence of any commercial or financial relationships that could be construed as a potential conflict of interest.
