# Supplementary material for: An Original Ferroptosis-Related Gene Signature Effectively Predicts the Prognosis and Clinical Status for Colorectal Cancer Patients
Source: Front Oncol. 2021 Jun 24;11:711776. doi: 10.3389/fonc.2021.711776 (PMC8264263; doi:10.3389/fonc.2021.711776)
Supplement: Supplementary file 1 [file DataSheet_1.pdf]

# Fig. S1

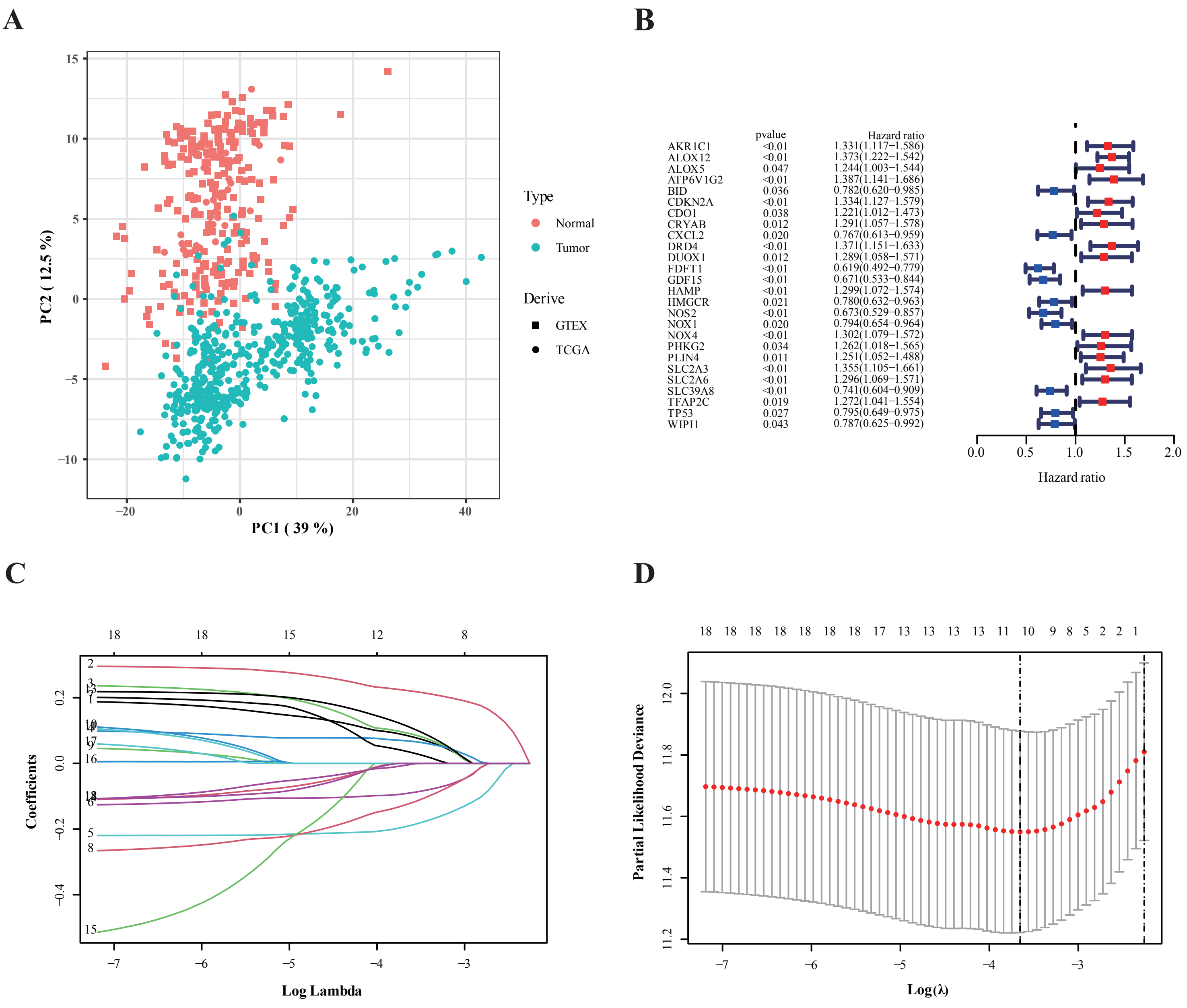


Fig.S1 PCA (A) plot between GTEx and TCGA datasets. Forest plot (B) to show the results of the univariate cox regression analysis between ferroptosis-related gene expression and prognosis. Figure (C) to show the Log Lambda value corresponding to the minimum cross-validation error point. Figure (D) to show the prognostic ferroptosis-related genes with non-zero coefficient corresponding to the same Log Lambda value were selected for subsequent signature construction.

# Fig. S2

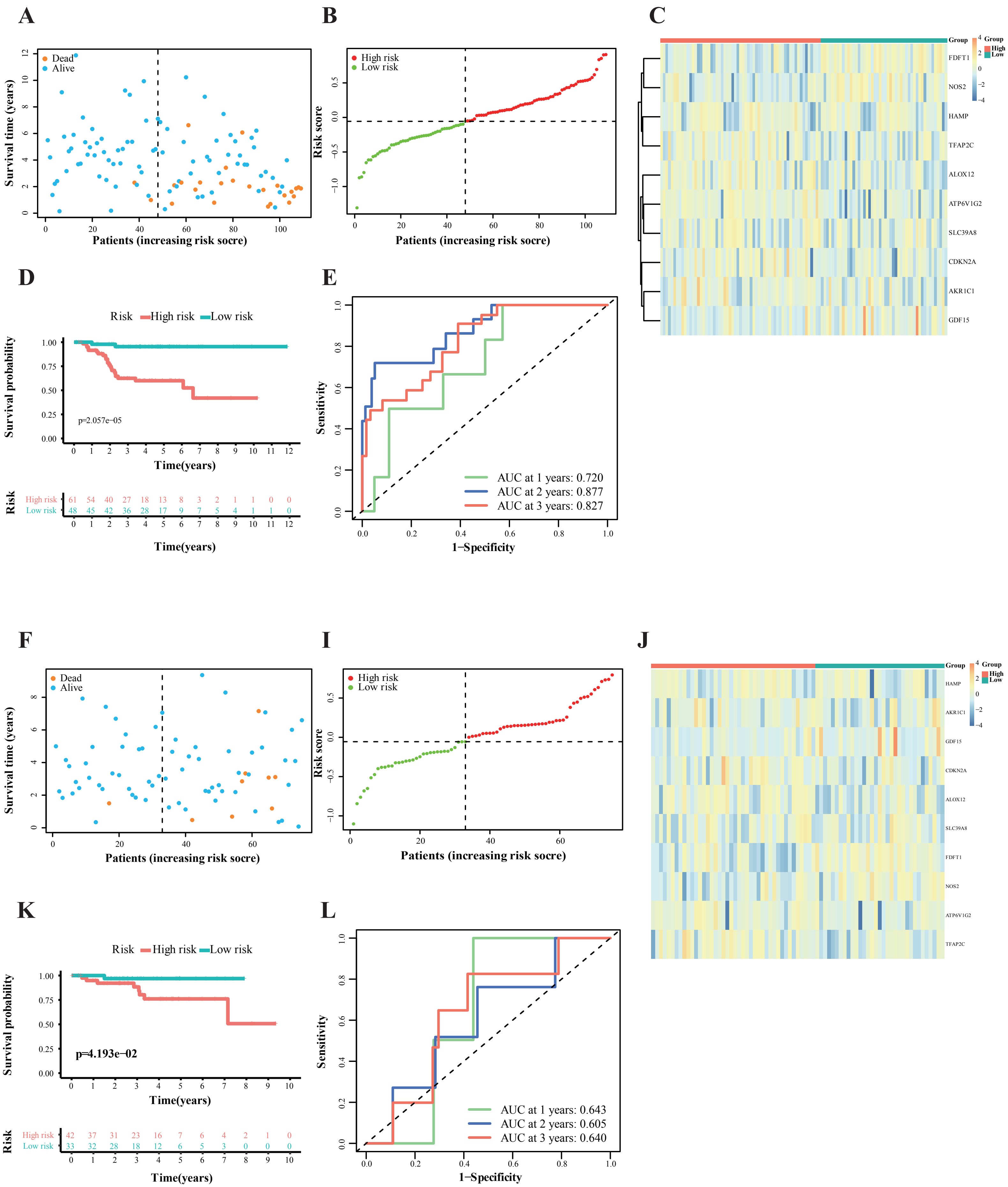


Fig. S2 The OS status plots, OS and risk score plots, heatmaps, Kaplan–Meier survival plots and ROC plots of these 10 genes between left-hemi (A-E) and right-hemi (F-L) CRC patients in the GSE14333 dataset.

# Fig. S3

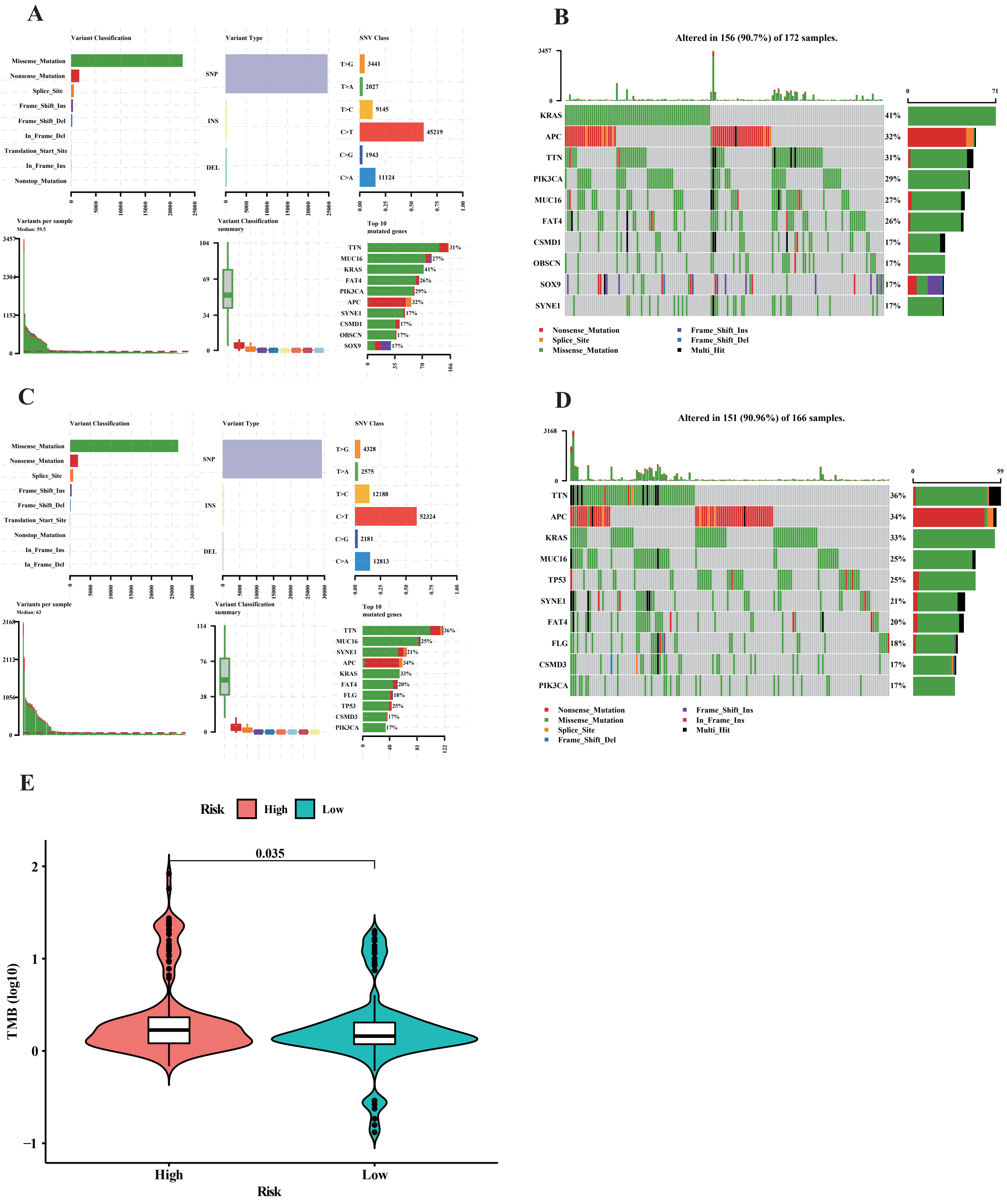


Fig. S3 The maf-summary plots and oncoplots of thesomatic mutation and the violin plot (E) for the TMB scores between the high-risk (A-B) and low-risk (C-D) groups in the ICGC dataset.

# Fig. S4

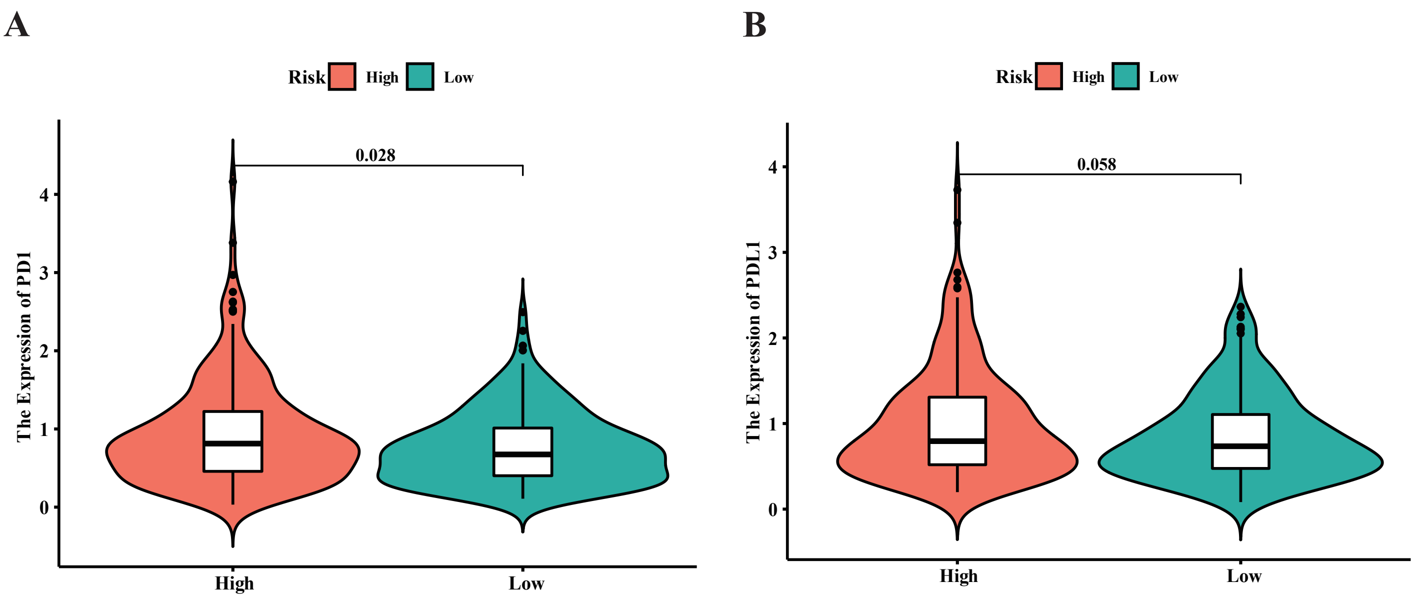


Fig. S4 The violin plots for the relationships between the risk scores and the expressions levels of the PD-1 (A) and PD-L1 (B) in the TCGA dataset.
